# Degradation kinetics of α‐conotoxin TxID

**DOI:** 10.1002/2211-5463.12697

**Published:** 2019-08-02

**Authors:** Pan Xu, Yang Xiong, Yiqiao Liu, Shurun Yu, Dongting Zhangsun, Yong Wu, Sulan Luo

**Affiliations:** ^1^ Key Laboratory of Tropical Biological Resources of Ministry of Education Key Lab for Marine Drugs of Haikou School of Life and Pharmaceutical Sciences Hainan University Haikou Hainan China

**Keywords:** activity assay, stability and degradation kinetics, UPLC, α3β4 nAChR, α‐Conotoxin TxID

## Abstract

α‐Conotoxin (CTx) TxID is a potent α3β4 nicotinic acetylcholine receptor (nAChR) antagonist that has been suggested as a potential drug candidate to treat addiction and small cell lung cancer. The function and structure of TxID have been well‐studied, but analyses of its stability have not previously been reported. The purpose of this study was to analyze the stability and forced degradation of TxID under various conditions: acid, alkali, water hydrolysis, oxidation, light, thiols, temperature, ionic strength and buffer pH. Different degradation products were formed under various conditions, and the degradation patterns of TxID showed pseudo‐first‐order kinetics. TxID degraded slowest at pH 3 within a pH range of 2–8. The major degradation products were analyzed using liquid chromatography–tandem mass spectrometry and the activity of the main product with α3β4 nAChR was analyzed using electrophysiological methods. Our analysis of TxID stability may aid the selection of appropriate conditions for peptide production, packaging and storage.

**Abbreviations**
ACN: acetonitrile
CTx: conotoxin
ESI: electrospray ionization
GSH: reduced glutathione
HSA: human serum albumin
ICH: International Conference on Harmonisation
LC: liquid chromatography
MS/MS: tandem mass spectrometry
nAChR: nicotinic acetylcholine receptor
RSD: relative standard deviation
RT: retention time
*t*_0.5_: half‐life period
*t*_0.9_: validity period
TFA: trifluoroacetic acid
UPLC: ultra‐performance liquid chromatography

Nicotinic acetylcholine receptors (nAChRs) are widely distributed throughout the central and peripheral nervous systems. They are important in normal physiology and have been found to be involved in many diseases 1, such as epilepsy, pain, addiction, Alzheimer's disease, Parkinson's disease, schizophrenia, and breast and lung carcinoma 2, 3, 4, 5, 6, 7. nAChRs are assembled as pentamers from α (α1–α7, α9, α10) and/or β (β2–β4) subunits to form a variety of subtypes in mammalian cells 8, 9. The α3β4 nAChR subtype is mainly expressed in the medial habenula of the central and peripheral nervous systems. It is the most prevalent subtype in the brain and is associated with nicotine addiction 10, neuropathic pain 11 and small cell lung cancer 12. Ligands of α3β4 nAChR have certain effects in tobacco and alcohol addiction, and it is expected to become a new target for treating addiction 13.

α‐Conotoxins (α‐CTxs) were first obtained from the venom of cone snails and specifically inhibit various nAChR subtypes 14, 15. They can be developed as useful probes to detect the mechanism of ligand–nAChR interaction and to study specific physiological functions of nAChRs 16, 17.

α‐Conotoxin TxID, which blocks the rat α3β4 nAChR subtype with an IC_50_ of 12.5 nm, was discovered in our lab. It is one of the most potent α3β4 nAChR antagonists to date 18. α‐CTx AuIB, which also showed an effect on α3β4 nAChRs, has a lower potency but can reduce mechanical allodynia, suggesting that CTx acting on α3‐containing nAChRs may have a role in the treatment of neuralgia 11. These studies suggested that TxID may have possibilities for the treatment of nicotine addiction, neuralgia, etc.

Previous studies mainly focused on the structure and activity of TxID, while its stability remains unknown. Peptides are considered to be unstable and have multiple degradation pathways under various conditions 19, such as light exposure, oxidation, temperature and pH. An investigation of stability would help to elucidate degradation mechanisms and pathways of the peptide 20. Through these studies, it should be possible to select suitable storage conditions for the peptide to improve its stability 21. Such studies will also help in understanding changes in the purity and characteristics of the peptide 22.

The purpose of this work was to (a) determine the stability of TxID under International Conference on Harmonization of Technical Requirements for Registration of Pharmaceuticals for Human Use (ICH) recommended conditions, (b) assess the degradation kinetics of TxID, and (c) identify the main degradation products using liquid chromatography (LC)–tandem mass spectrometry (MS/MS) analysis.

## Results and Discussion

### Chemical synthesis and oxidative folding of TxID isomers

**Figure 1:**
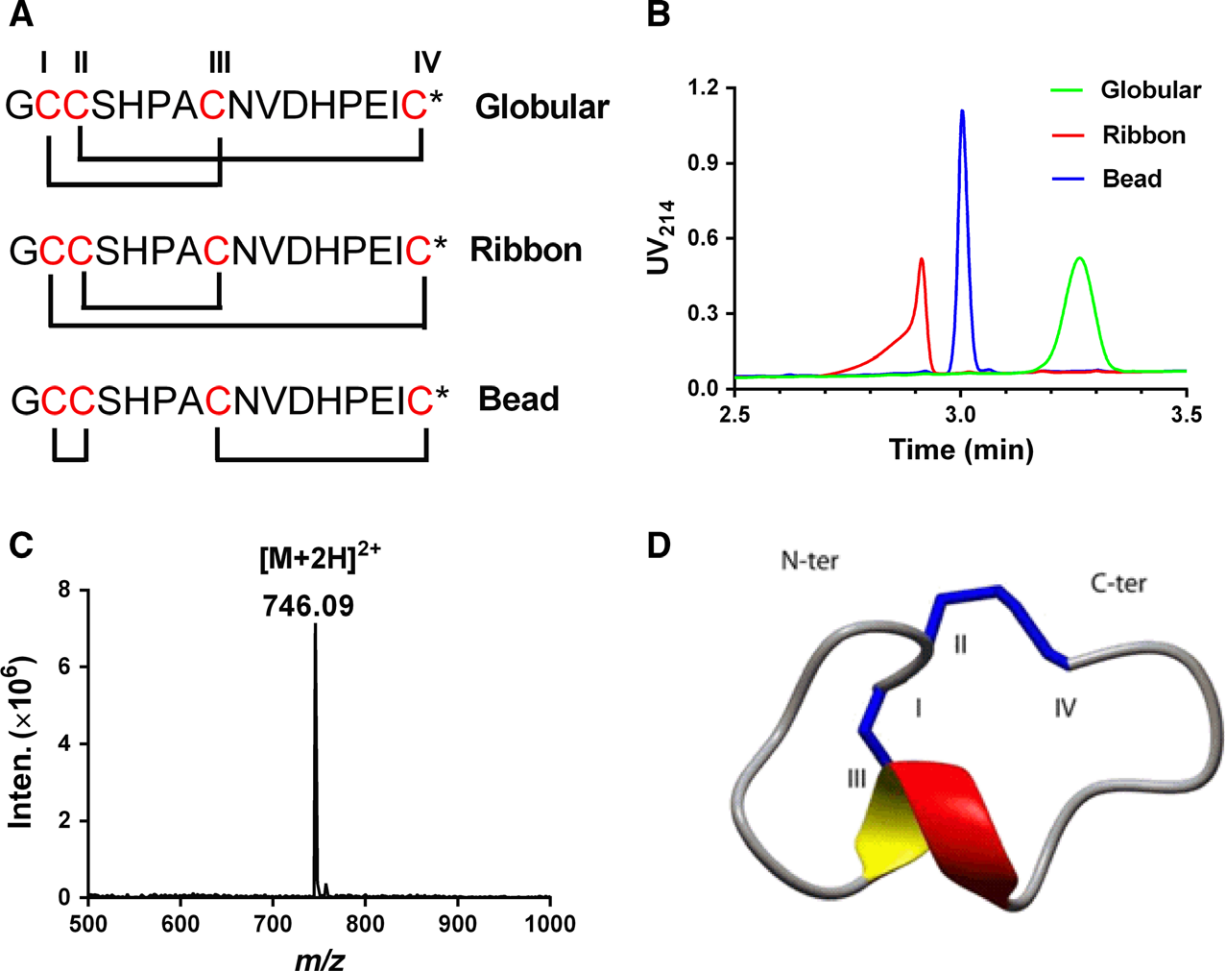
UPLC, ESI‐MS and structure profile of TxID. (A) Schematic representation of globular (native), ribbon and bead isomer disulfide connectivities of TxID. Asterisks represent amidated C terminus. (B) Overlay of UPLC traces for globular isomer (green), ribbon isomer (red) and bead isomer (black). (C) ESI‐MS data for globular isomer. (D) NMR analysis showing the 3D structure of TxID. Inten, Intensity.

Three isomers of TxID were synthesized by the regioselective two‐step oxidation method as described in ‘Materials and methods’. The bridging pattern of CysI–III and CysII–IV is that of native TxID (globular isomer), and alternative connectivity CysI–IV and CysII–III gives a ribbon isomer and CysI–II and CysIII–IV gives the bead isomer (Fig. 1A). After each folding step, the final oxidized peptide was purified by preparative reverse‐phase HPLC. The co‐elution of the three isomers is shown in Fig. 1B, where the globular isomer has the longest retention time (RT). The observed molecular mass (1489.8 Da) of the final oxidation product of the globular isomer is consistent with the calculated mass (1490.1 Da) (Fig. 1C). Luo *et al*. 18 obtained the three‐dimensional solution structure of the globular isomer of TxID by NMR, and this, generated using the program molmol, is shown in Fig. 1D.

### Validation

This result is a verification of the ultra‐performance liquid chromatography (UPLC) quantification method. The linearity of an analytical procedure is its ability to obtain test results that are directly proportional to the concentration of the analyte in the sample. TxID showed linearity in the tested concentration range of 1–100 μg·mL^−1^. A very high correlation coefficient (*r* ^*2*^) of 0.9999 was obtained. The equation of the line was *y* = 22808*x *− 66473. This equation was used to determine the amount of TxID present in a stable sample. For UPLC evaluation, the intraday and interday accuracies are shown in Table 1. The relative standard deviation (RSD) for intraday precision was found to be in the range of 0.58–1.20%, whereas the RSD for interday precision was in the range of 0.76–3.30%. Also, good recoveries were obtained at three concentrations.

**Table 1:** Intraday and interday precision and accuracy (%) of standards at 5, 40, 80 μg·mL^−1^ of TxID. Conc, concentration. RSD (%): SD/mean × 100.

Theoretical Conc (μg·mL^−1^)	Intraday (*n* = 3)			Interday (*n* = 5)		
	Mean Conc ± SD (μg·mL^−1^)	RSD (%)	Accuracy (%)	Mean Conc ± SD (μg·mL^−1^)	RSD (%)	Accuracy (%)
5	5.06 ± 0.06	1.20	100.5 ± 0.010	4.98 ± 0.16	3.30	99.60 ± 0.03
40	39.35 ± 0.23	0.58	98.4 ± 0.006	40.72 ± 1.17	2.80	101.80 ± 0.03
80	81.48 ± 0.60	0.73	101.8 ± 0.007	79.96 ± 0.01	0.76	99.90 ± 0.007

### Stability and major degradation products

#### Influence of light and oxidation conditions

**Figure 2:**
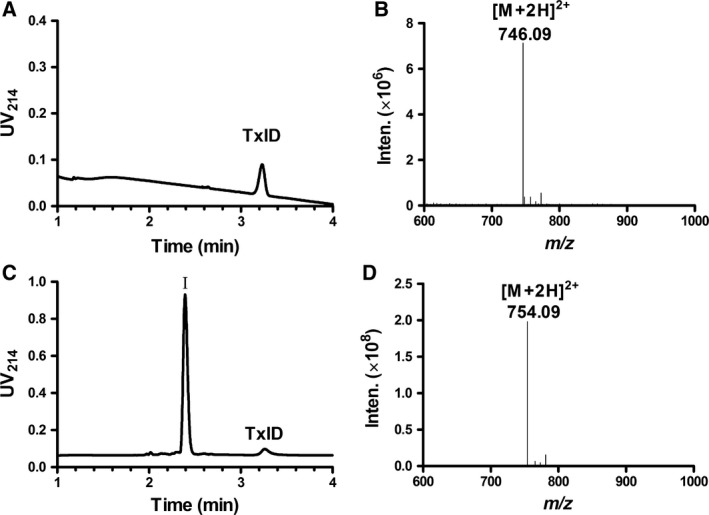
UPLC chromatograms and mass spectra of TxID. (A) light condition. (B) Electrospray ionization mass spectrometry (ESI‐MS) of TxID. (C) 30% H_2_O_2_ condition. (D) ESI‐MS data for degradation product I of TxID in 30% H_2_O_2_.

In both wrapped and unwrapped states, light had no significant effect on the stability of TxID in solid form under UV light. There were no degradation products formed from photolytic degradation (Fig. 2A).

**Figure 3:**
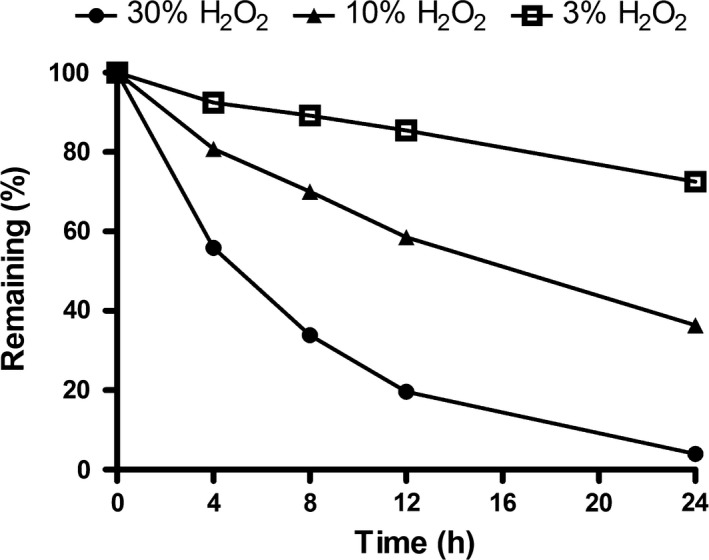
TxID stability in oxidative conditions of three different concentrations of H_2_O_2_ (3%, 10%, 30%) at room temperature. Data points are means ± SEM of three experiments (*n* = 3). The error bars are not visible because they are smaller than the symbols.

**Figure 4:**
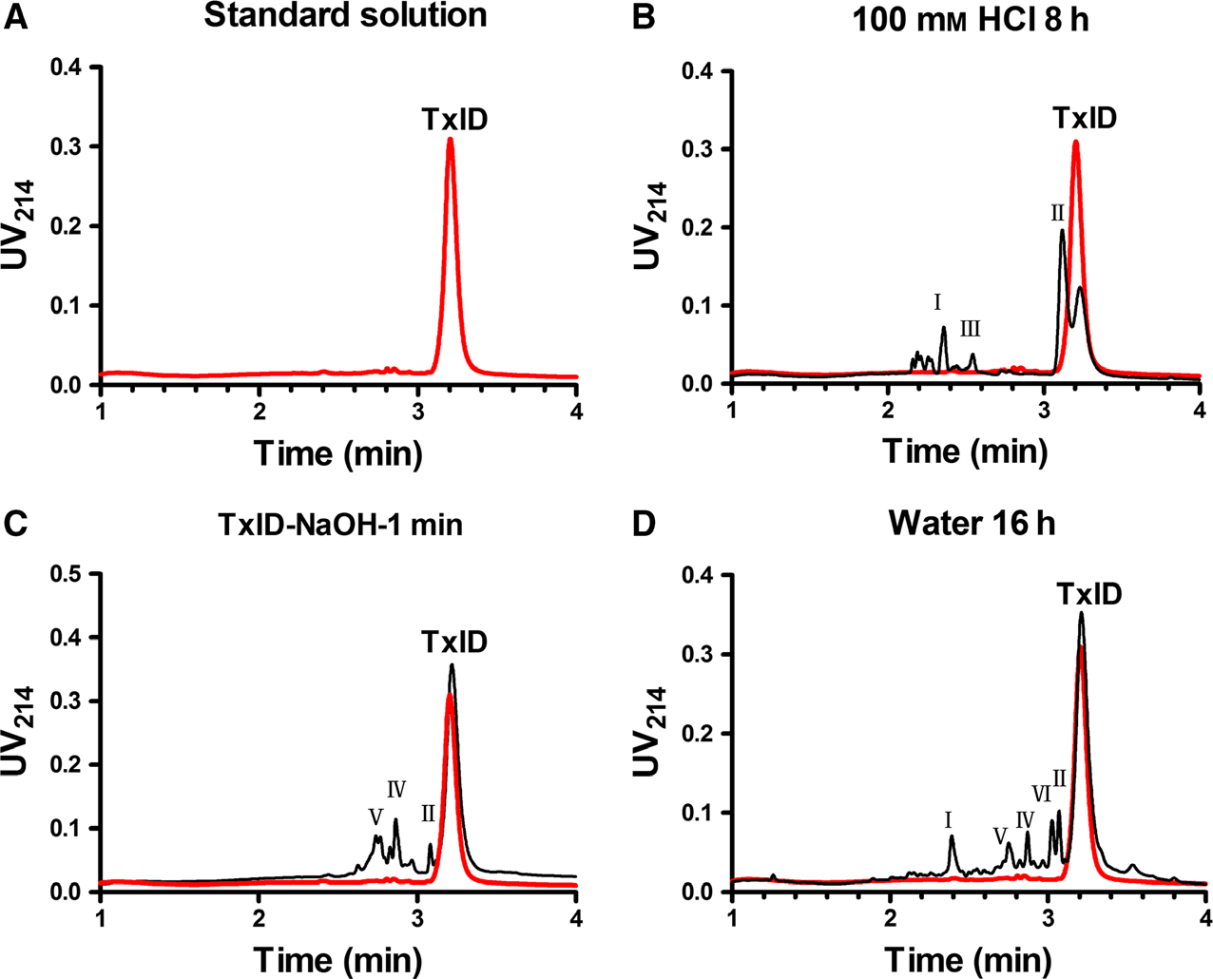
Chromatograms showing the degradation behavior of TxID and unknown products at different times (red lines represent samples at time 0) under various stress conditions: (A) standard solution of TxID; (B) 100 M HCl; (C) 100 M NaOH; and (D) water.

The stability of TxID in 3%, 10% and 30% H_2_O_2_ during 24 h is shown in Figs 3 and 4 and Table 2. The remaining amount of TxID decreased with increasing H_2_O_2_ concentration and incubation time extension (Table 2, Fig. 3).

**Table 2:** Effects of various stress conditions on stability of TxID.

Stress condition	Time	TxID remaining (%)	Incubation temperature (°C)	Product no.: retention time (min)

Light: 4500 Lx	7 days	95	25	—
Oxidation				
3%, v/v H_2_O_2_	24 h	85	25	I: 2.37
10%, v/v H_2_O_2_		58		
30%, v/v H_2_O_2_		7		
Acidic: 100 mM	8 h	36	80	I: 2.37; II: 3.10; III: 2.53
Alkaline: 100 mM	1 min	71	80	II:3.10; IV:2.86; V:2.75
Water	16 h	63	80	I: 2.37; II: 3.10; IV:2.86; V:2.75; VI: 3.02

There was only one degradation product (I) of TxID in the incubation in H_2_O_2_ for 24 h, which was the peak with 2.37 min RT in Fig. 2C. It showed the precursor (*m/z* 746.09 (M+H)^+^) and fragment ions (*m/z* 754.09) with observed molecular mass of 1506.18 Da (Fig. 2D), which had a mass shift of 16 Da compared with native TxID with molecular mass of 1490.18 Da (Fig. 2B). The addition of oxygen to TxID when it was incubated in H_2_O_2_ was clearly indicated. TxID degraded in the presence of oxygen owing to the methionine (Met) in its sequence. The Met of TxID was oxidized in H_2_O_2_. The side chain group of Met has a sulfur atom, which is susceptible to electron transfer oxidation to give sulfoxide 23. When Met was oxidized to form methionine sulfoxide in TxID, the inhibition on α3β4 nAChR decreased 13‐fold in potency compared to native TxID 24. Some researchers have suggested that adding antioxidants to react with oxidants could prevent degradation 25.

#### Influence of acidic, alkaline and neutral conditions

**Figure 5:**
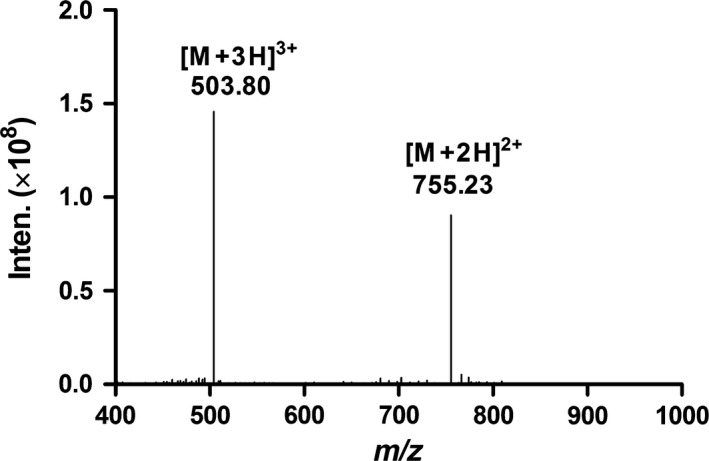
ESI‐MS data for degradation product II of TxID in 100 mm HCl and NaOH. Inten, Intensity.

The chromatogram of TxID standard solution showed a peak with RT of 3.19 min (Fig. 4A). Under strong acidic conditions, TxID was degraded to three major products, i.e. I, II and III (Table 2, Fig. 4B). A significant amount of degradation product II was observed with a molecular mass of 1508.4 Da, which was 18 Da more than that of the native TxID (Fig. 5). We hypothesized that one of the amide bonds in TxID might have undergone hydrolytic cleavage in which the carbonyl group plus a hydroxide ion had become a carboxyl group and the amino group plus a hydrogen ion had become an amino group, while disulfide bonds remained intact.

TxID degraded much more rapidly in the alkaline solution than in acidic conditions. In 100 mm NaOH solution, TxID was rapidly degraded during sample preparation. The main degradation products were II, IV and V, in which product II had the same RT as that in acidic conditions (Table 2, Figs 4C and Fig. 5).

Compared with acidic and alkaline conditions, degradation of TxID in water for 16 h occurred at a slower rate, forming five main products I, II, IV, V and VI (Fig. 4D). There were two products with the same RT and molecular mass observed both in water and in alkaline conditions (Table 2, Fig. 4D). Because the amount of degradation products III, IV, V and VI was very small, their molecular masses were not detected.

#### Thiol stability

**Figure 6:**
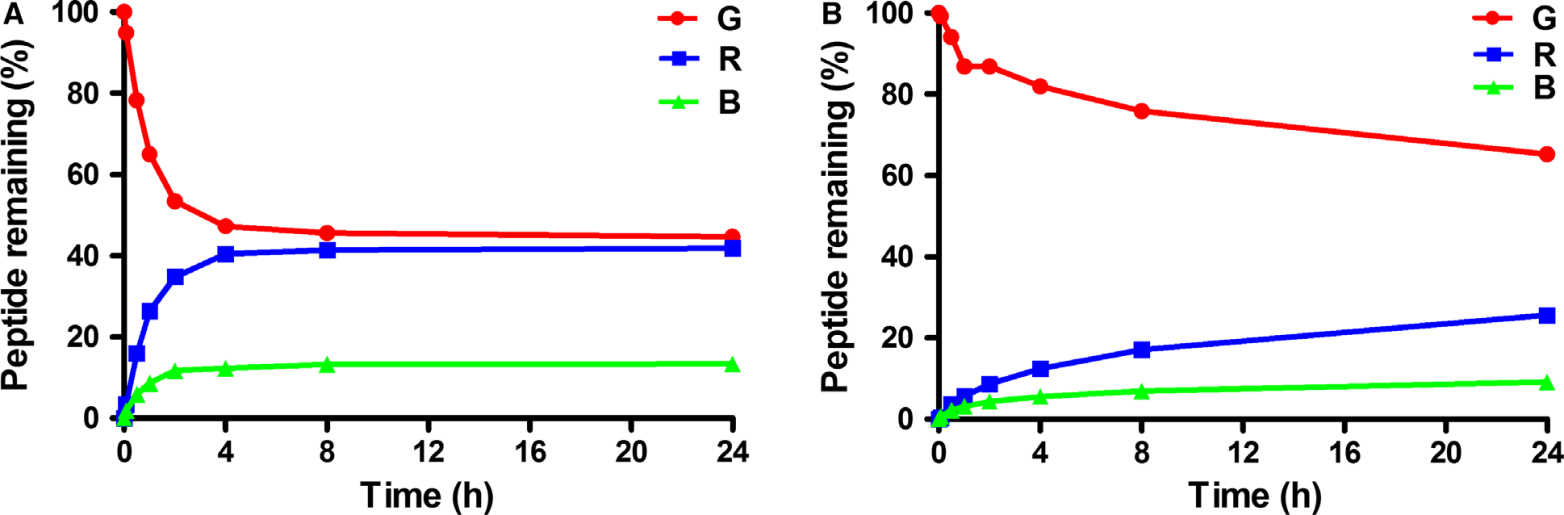
Scrambling analysis of TxID's disulfide bonds. (A) In GSH solution. (B) In HSA solution. G, R and B represent globular, ribbon and bead isomers of TxID, respectively. Data points are means ± SEM of three experiments (*n* = 3). The error bars are not visible because they are smaller than the symbols.

TxID was dissolved in reduced glutathione (GSH) solution to investigate the effect of external thiols on the scrambling of the TxID disulfide framework. Under this condition, the disulfide bond framework of the globular TxID isomer was scrambled immediately, as shown in Fig. 6A. The globular isomer underwent GSH‐assisted shuffling. About 50% of globular TxID remained after 4 h of incubation, and in addition, the ribbon and bead isomers were produced subsequently and increased to 40% and 10% of the total, respectively. The final content of the three isomers tended to balance (Fig. 6A).

Albumin is the main component of plasma proteins, usually present at more than 50% 26. It can disrupt disulfide bonds significantly in peptides that contain them, such as AM‐336 and human atrial natriuretic peptide 27. Similarly, TxID was shown to undergo disulfide scrambling in human serum albumin (HSA) solution (Fig. 6B). After a 6 h incubation, the globular isomer remaining was 75% of the total, and the ribbon and bead isomers were 20% and 5%, respectively (Fig. 6B). Among all the conditions, the production of the bead isomer was the lowest. In a thiol‐containing environment, disulfide bonds are kinetically unstable 28. Therefore, the stability of disulfide bonds in α‐CTx can be evaluated using a thiol‐containing molecule, such as glutathione or serum albumin 27.

### Degradation kinetics of TxID

#### Influence of pH

**Figure 7:**
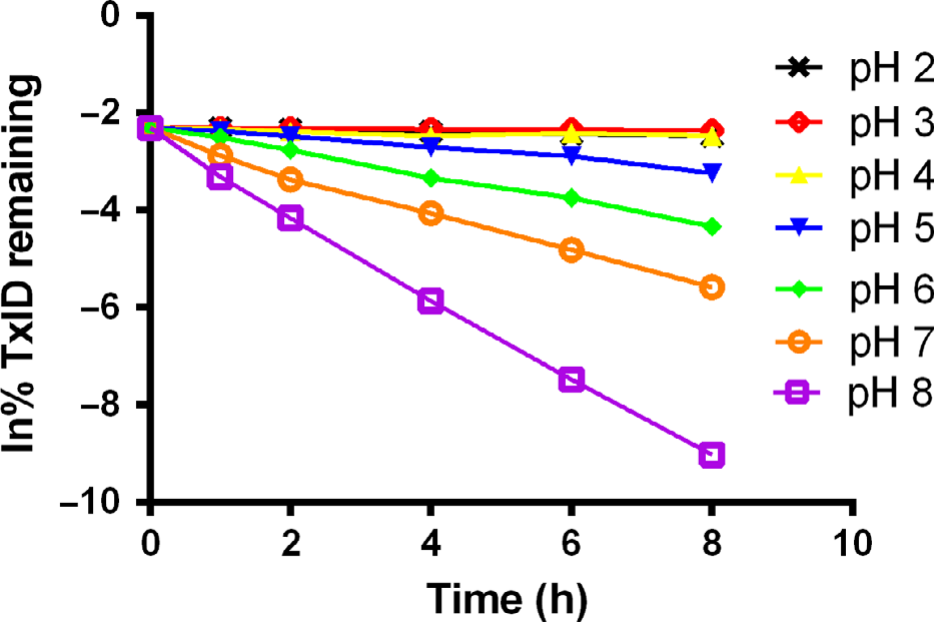
First‐order plots showing degradation of TxID at 80 °C in pH 2, 3, 4, 5, 6, 7 and 8 buffers. Data points are means ± SEM of three experiments (*n* = 3). The error bars are not visible because they are smaller than the symbols.

**Figure 8:**
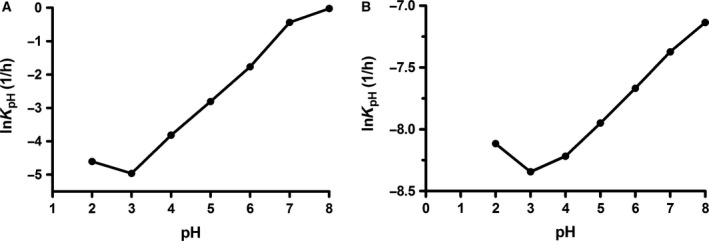
The pH–rate (*K*_obs_) profiles of TxID degradation. (A) Reaction at 80 °C. (B) Reaction at 25 °C (room temperature). Data points are means ± SEM of three experiments (*n* = 3). The error bars are not visible because they are smaller than the symbols.

The high linearity (*r *>* *0.99) of the change in concentration *vs* time, and the degradation of TxID followed first‐order kinetics within the pH range of 2.0–8.0 at 80 °C (Fig. 7). Based on the V‐shaped pH–rate curve, the natural logarithm of *K*_obs_ was plotted against pH in Fig. 8. It can be seen from Fig. 8A,B that the trends of the V‐shaped pH–rate profiles at the two temperatures (80 and 25 °C) are similar, which indicated that TxID was stable at pH 3.0 in potassium phosphate buffer. The results showed that the catalytic hydrolysis rate was much faster in alkaline than in acidic condition. At pH 3.0, the reaction rate was roughly 14 times slower than that at pH 2.0, indicating that TxID is more stable than at pH 2.0. It showed a stability 280 times higher than at pH 8.0. In other words, TxID is relatively more stable in acidic conditions than in alkaline conditions.

The catalytic rate constants of H^+^ and OH^−^ ions (*K*_H_ and *K*_OH_) of TxID in aqueous solutions were 5.1 and 39 000, respectively (Table 3). *K*_OH_ was 7781 times higher than *K*_H_. This indicated that hydrolysis of TxID by OH^−^ was much more complete than that by H^+^. Since TxID has different degradation rates under the three hydrolysis conditions, different degradation products were observed in Fig. 4.

**Table 3:** Catalytic rate constants and degradation rate constants of TxID.

Catalytic rate constants (1/Mh)		Degradation rate constant at four Temperatures (1/Mh)				Degradation rate constant at three ionic strengths (1/Mh)		
*K*_H_	*K*_OH_	*K*_obs_ (50 °C)	*K*_obs_ (60 °C)	*K*_obs_ (70 °C)	*K*_obs_ (80 °C)	*K*_obs_ (0.3 m)	*K*_obs_ (0.5 m)	*K*_obs_ (0.7 m)
5.01	3.90 × 10^4^	1.01 × 10^−3^	2.42 × 10^−3^	5.54 × 10^−3^	2.02 × 10^−2^	5.06 × 10^−2^	5.07 × 10^−2^	5.07 × 10^−2^

#### Influence of temperature

**Figure 9:**
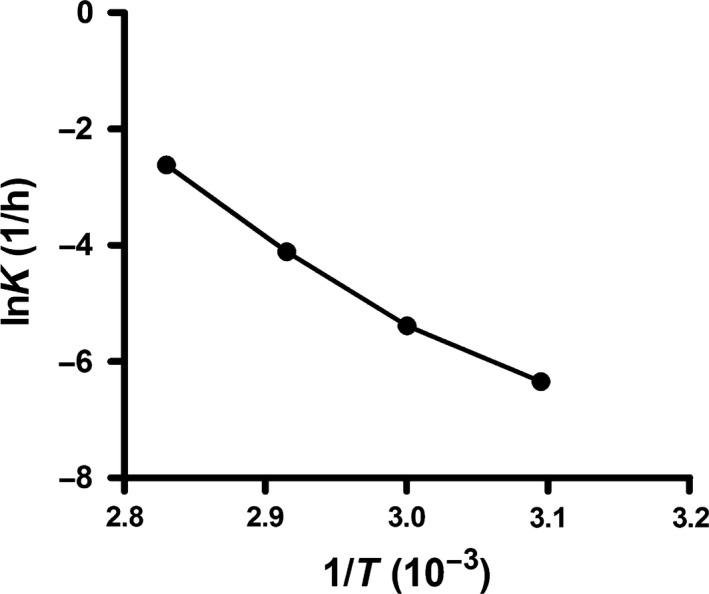
Arrhenius plot for the degradation rate constant of TxID in aqueous solution. Data points are shown as means ± SEM of three experiments (*n* = 3). The error bars are not visible because they are smaller than the symbols.

The most stable pH value was 3.0’. Therefore, TxID stability was evaluated at four temperatures (50, 60, 70 and 80 °C) with pH 3.0 buffer. It was clear that when the temperature increased by 10 °C, the degradation rate increased by at least two‐fold (Table 3). An Arrhenius plot was constructed with high linearity (*r* > 0.99, Fig. 9). The degradation rate increased with temperature. High temperature accelerated the reaction. The *E*_a_ value can provide a theoretical basis for predicting the half‐life period (*t*_0.5_) and validity period (*t*_0.9_) of TxID solution at 25 °C, which were 707 and 107 days, respectively (Table 4).

**Table 4:** Activation energy and shelf lives of TxID in aqueous buffer with pH 3.0.

pH	*A* (1/h)^a^	*E*_a_ (kJ·mol^−1^)^b^	$t_{0.9}^{25^{\circ }C}$ c	$t_{0.5}^{25^{\circ }C}$ d
3	1.22 × 10^16^	117.086	107	707

^a^Frequency factor. ^b^Activation energy. ^c^Validity period at 25 °C. ^d^Half‐life period at 25 °C.

#### Influence of ionic strength

The ionic strength had no significant effect on stability of TxID (Table 3). Previous research has shown that salt ions may have an effect on the electrostatic interactions of peptides 29. At low salt concentration, there was reduced attraction of heterogeneous charges and repulsion of the same charge within the peptide. TxID was stable in neutral NaCl solution. With increasing NaCl added, TxID stability was not affected by the charge–charge interactions.

### Activities of degradation products of TxID on the α3β4 nAChR

**Figure 10:**
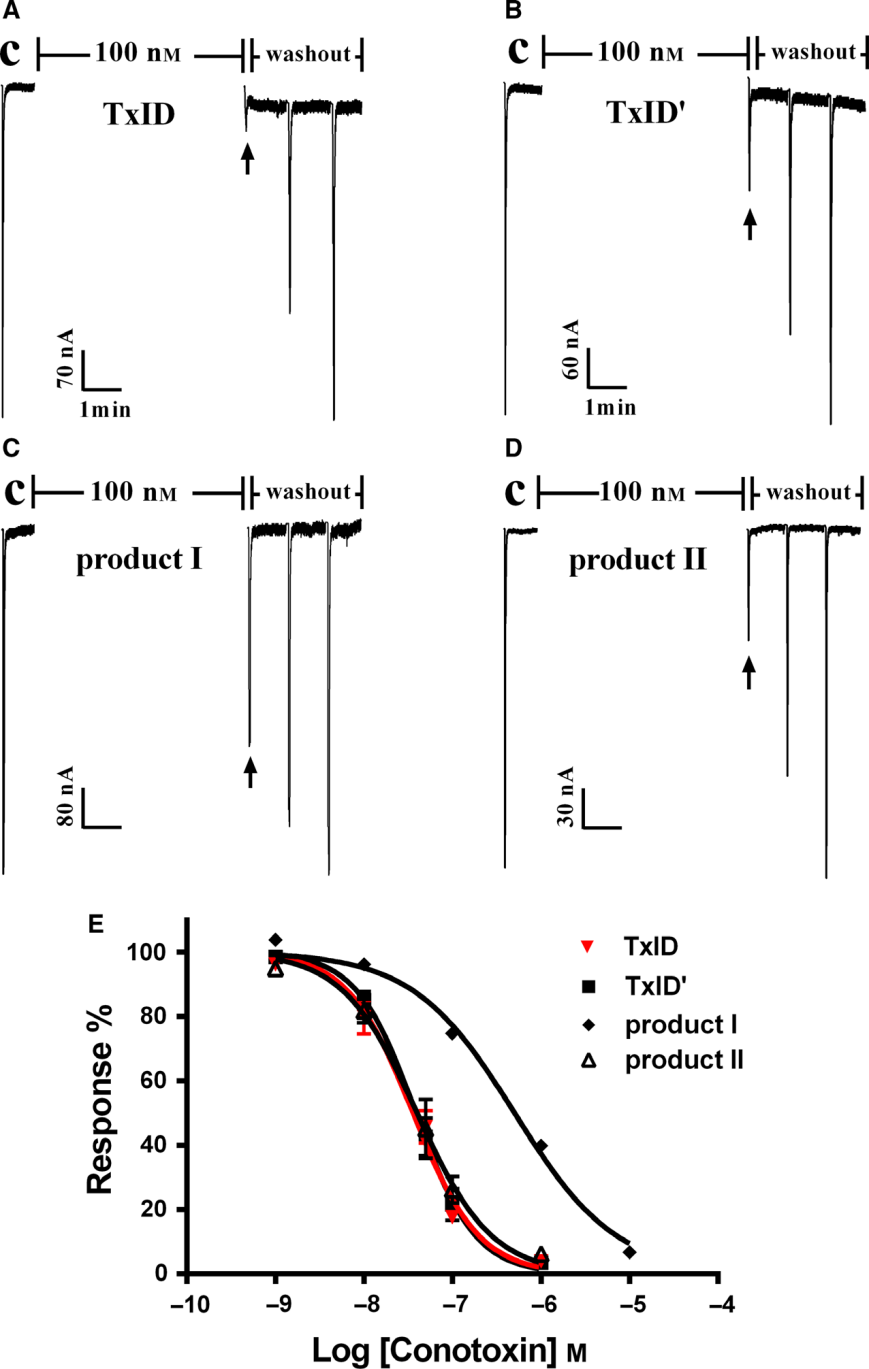
Activity of TxID and it degradation products at α3β4 nAChR expressed in *Xenopus* oocytes. Oocytes were voltage‐clamped at −70 mV, and current responses were induced by 1 s 100 μm ACh. (A) TxID at 100 nm. (B) TxID′ at 100 nm. (C) Product I at 100 nm. (D) Product II at 100 nm. (E) Concentration‐dependent response curves of α3β4 nAChR. Values are mean ± SEM from three to four separate oocytes.

To investigate whether the remaining TxID (TxID′) observed by the hydrolysis reaction exhibits the same inhibitory activity as the parent TxID, we purified the TxID after hydrolysis by HPLC to test with α3β4 nAChR. At the same time, we also tested the two most abundant impurity products, product I and product II, purified by HPLC. As shown in Table 5 and Fig. 10E, both TxID′ and product II displayed similar inhibitory activities to native TxID. Though there was about 14‐fold decrease in the IC_50_ value of product I relative to native TxID, the result was similar to that reported previously 24. Representative traces of these four peptides on α3β4 nAChR are shown in Fig. 10. Product I was only potent with 15% response at 100 nm (Fig. 10C) and the other three peptides blocked α3β4 nAChR with 24–29% response at 100 nm (Fig. 10A,B,D). These results indicated that the cleavage of the amide bond in TxID has no effect on the activity of the α3β4 nAChR subtype, but the oxidation of Met affects the binding activity of this subtype. Ren *et al*. 24 used molecular dynamics simulations to show that the Met residue can establish a hydrophobic interaction with the residues in the binding pocket; when Met is oxidized, the hydrophobic interaction between the peptide and the receptor is weakened, resulting in decreased binding activity to α3β4 nAChR.

**Table 5:** Effect of TxID and its degradation products on α3β4 nAChR expressed in *Xenopus* oocytes.

Toxin	IC_50_ (nm)^a^	Hill slope^a^	Ratio^b^
TxID	35.9 (28.9–44.7)	1.17 (0.87–1.46)	1
TxID′	38.7 (32.5–46.2)	1.29 (0.99–1.59)	1.1
Product I	503.4 (412.9–613.7)	0.76 (0.65–0.86)	14.0
Product II	39.8 (31.8–49.9)	1.03 (0.89–1.27)	1.1

^a^IC_50_ values with 95% confidence interval. ^b^Indicates the ratio of IC_50_ values relative to TxID.

## Conclusions

In conclusion, this study was an analysis by UPLC based on ICH recommendations. The analytical method used to evaluate the stabilities of TxID under various conditions was precise and accurate. The results showed that (a) temperature, pH and an oxidative environment affected the stability of TxID significantly; (b) the TxID disulfide framework was influenced obviously by external thiols; (c) there was no impact of ionic strength and light; (d) based on mass spectrometry analysis, two main degradation products of TxID were identified: TxID′ and product II had similar activity to the native TxID, while activity of product I decrease 14‐fold with α3β4 nAChR; and (e) the degradation of TxID in the aqueous system followed first order kinetics. Assessing the impact of different factors on TxID stability would help to select the right conditions for peptide production, packaging and storage.

## Materials and methods

### Material

TxID linear peptide was synthesized by GL Biochem Co. Ltd (Shanghai, China). Water was from ELGA (Lane End, High Wycombe, UK). Trifluoroacetic acid (TFA) and acetonitrile (ACN) were HPLC grade, obtained from Tedia Company (Fairfield, OH, USA) and Thermo Fisher Scientific (Waltham, MA, USA), respectively. Diethyl ether, phenol, and triisopropylsilane were from Tedia Company Reduced glutathione was from Sigma (St. Louis, MO, USA) . Other reagents were analytical grade.

### Instrumentation and chromatographic conditions

A UPLC system along with Acquity™ UPLC BEH 300 C_18_ column (1.7 μm, 2.1 × 50 mm) at 40 °C was used for analysis of TxID, and was equipped with a Waters Acquity UPLC photodiode array detector, a Waters Acquity UPLC class sample manager (set at 4 °C), and a Waters Acquity UPLC quaternary solvent manager (Milford, MA, USA). empower 3.0 software was developed by Waters for data acquisition (Milford, MA, USA). The linear gradient program was as follows: 0–3.5 min 10–40% buffer B. The wavelength was monitored at 214 nm, and the flow rate was at 0.5 mL·min^−1^. The injection volume was 10 μL. Buffer A was 0.65% TFA in water, and buffer B was 0.5% TFA in 90% ACN and 10% water, degassed through sonication before use.

A Waters XEVO TQD equipped with an electrospray ionization (ESI) source is a triple‐quadrupole tandem mass spectrometer detector.

The optimal MS parameters were cone voltage of 28 V, capillary voltage of 0.5 kV, and desolvation temperature of 600 °C. The desolvation flow rate was 800 L·h^−1^ and the cone flow was 50 L·h^−1^. The collision voltages was 3 V.

masslynx software version 4.1 was developed by Waters for data acquisition and instrument control (Milford, MA, USA).

### Peptide synthesis and folding

All peptides were synthesized by solid‐phase methodology using Fomc (*N*‐(9‐fluorenyl) methoxycarbonyl) chemistry and standard side‐chain protection, except for cysteine residues, which were protected in pairs with either an *S‐*trityl group (S‐Trt) on CysI–III (globular), CysII–III (ribbon) and CysI–II (bead) or an *S‐*acetamidomethyl group (S‐Acm) on CysII–IV (globular), CysI–IV (ribbon) and CysIII–IV (bead). The three isomers of TxID were synthesized and purified as previously described 30. A Two‐step oxidation method was used to form two disulfides for each isomer of TxID. The first disulfide bond was performed in 20 mm potassium ferricyanide (K_3_[Fe(CN)_6_]) for 45 min at room temperature, and the monocyclic peptide was purified by reversed‐phase (RP)‐HPLC. The second disulfide bond was formed by removal of *S*‐Acm‐protected groups using iodine oxidation. The monocyclic peptide was dropped into an equal volume of iodine (10 mm) in water : TFA : ACN (73 : 3 : 24 by volume) and allowed to react for 5 min. The reaction was quenched by adding ascorbic acid, resulting in decolorization of the solution. Peptides was then purified by RP‐HPLC using a gradient of 10–50% buffer B for 30 min. Buffer A was 0.1% TFA in water and buffer B was 0.09% TFA in 90% ACN. Absorbance was monitored at 214 nm. The final purified peptides were analyzed and confirmed by RP‐UPLC and ESI‐MS. The active globular isomer of TxID with two disulfide bonds of Cys I‐III and CysII‐IV connectivities was used for all the following experiments.

### Stock solutions of TxID

TxID was dissolved in distilled water to obtain a stock solution with concentration of 1 mg·mL^−1^. Different concentrations of working solutions were prepared from the experimental stock solutions.

### Validation method

The validation method was performed in accordance with the ICH guidelines 31. The stock solution was diluted to six levels (1, 5, 10, 25, 50 and 100 μg·mL^−1^) for establishing a standard curve. The intraday and interday precisions were measured with three different concentrations (5, 40, 80 μg·mL^−1^) of solution on the same day and three consecutive days, respectively. All solutions were performed in triplicate and analyzed by UPLC by injecting 10 μL. The accuracy was evaluated with the above three different concentrations.

### Stability studies

Stability studies can help to identify the possible degradation products that help in understanding the pathway of degradation. All reactions were performed with a final concentration of 100 μg·mL^−1^ TxID, which was prepared by diluting stock solution with the appropriate experimental solutions. Forced degradation studies were conducted in accordance with the ICH Q1A (R2) guidelines 32, while drug and product light stability testing was in accordance with the ICH Q1B guidelines 33.

#### Influence of light

The degradation under light was performed by exposing the TxID powder in a photostability chamber with 4500 Lx for 1, 3, 5 and 7 days, respectively. The control samples were wrapped in light‐proof material to ensure that they remained in the dark in the same environment.

#### Influence of oxidation conditions

TxID was dissolved in H_2_O_2_ solution at three different concentrations of 3%, 10%, and 30% at room temperature. It was removed from the reaction mixture at 0, 1, 2, 4, 6, 8, 12 and 24 h sequentially for UPLC analysis.

#### Influence of acidic, alkaline and neutral conditions

TxID solution was prepared in an acidic condition (0.1 m HCl), an alkaline condition (0.1 m NaOH) and a neutral condition (water) and bathed at room temperature (~25 °C) or 80 °C. Aliquots were taken at appropriate times and immediately cooled with ice. The samples were analyzed by UPLC after neutralization and dilution. They were taken out of the reaction mixture at 0, 1, 2, 4, 6, 8, 12 and 24 h sequentially.

#### Thiol stability assay

Peptides were dissolved in a solution with 100 μL volume that contained 100 nm reduced glutathione (GSH) and HSA in 100 mm phosphate buffer plus 1 mm EDTA (pH 7.2). The samples were incubated at 37 °C. They were taken out of the reaction mixture at 0, 5 min, 1, 2, 4, 8 and 24 h sequentially and quenched immediately with 0.5% formic acid.

### Degradation kinetics examination

#### Influence of pH

The degradation rate constant (*K*_obs_) was determined with different pH values (pH 2.0–8.0) in 0.1 m PBS. The ionic strength (0.3 m) and temperature (25 and 80 °C) were constant. A pH meter was used to measure solution pH at room temperature. The stock solution of TxID was quickly added in a preheated buffer solution (80 °C). An aliquot was removed immediately to analyze the starting concentration (*C*_0_). The slope of the natural logarithmic (ln) plot of the remaining TxID score *vs* time (*t*) was in accordance with the following equation:

$$k_{\mathrm{obs}}=\left[\text{ln}\left(C_{0}-C_{t}\right)\right]/t$$

where the initial concentration of TxID is *C*_0_, and *C*_*t*_ is the remaining concentration at *t* time.

Catalytic rate constants were calculated according to the equation:

$$k_{\mathrm{obs}}=K_{0}+K_{H}^{+}+\left[{\text{H}}^{+}\right]+K_{{\mathrm{OH}}^{-}}\left[{\text{OH}}^{-}\right]$$

where the catalytic rate constant of the water molecules involved in the reaction is *K*_0_, $K_{H}^{+}$ and $K_{{\mathrm{OH}}^{-}}$ are the catalytic rate constants of H^+^ and OH^−^ ions, respectively.

#### Influence of temperature

The degradation rate of TxID was studied at 50, 60, 70 and 80 °C, respectively. An Arrhenius plot was used to analyze the effect of temperature on the degradation rate of TxID:

$$\text{ln}k_{\mathrm{obs}}=\text{lnA}-E_{a}/\left(\text{RT}\right)$$

where *A* is the frequency factor, *E*_a_ is the activation energy (J·mol^−1^), *R* is the universal gas constant (8.314 J·K^−1^·mol^−1^) and *T* is the absolute temperature (K). The half‐life period of degradation (*t*_0.5_) was calculated according to the equation:

$$t_{0.5}=0.693/k_{\mathrm{obs}}$$

The *t*_0.9_ was used as follows Eq:

$$t_{0.9}=0.1054/k_{\mathrm{obs}}$$

#### Influence of ionic strength

Ionic strength solutions were adjusted to 0.3, 0.5 and 0.7 m with NaCl. To investigate the effect on TxID of different ionic strengths (μ) in distilled water at 80 °C, ionic strength was calculated according to the equation:

$$\mu =[\sum \left(m_{n}\times z_{n}^{2}\right)]/2$$

where *m* represents the molar concentration of ion, *z* is the charge of ion, and *n* is the kind of ion.

The modified Debye–Huckel equation was used to evaluate the effect of μ on the degradation rate on TxID:

$$\text{log}k_{\mathrm{obs}}=\text{log}k_{0}+\alpha Z_{A}Z_{B}\frac{\surd \mu }{1+\surd \mu }$$

where *k*_obs_ is the observed rate constant, *k*_0_ is the rate constant at zero ionic strength, α is a constant for the solvent at a given temperature (α = 1.026 at 30 °C), and *Z*_A_ and *Z*_B_ are the charges on reactants A and B, respectively.

### Electrospray ionization mass spectrometry

The degradation products of TxID were analyzed using the UPLC and UPLC‐MS/MS. The chromatographic conditions for UPLC‐MS/MS study were the same as those for the UPLC method. The UPLC‐MS/MS study was carried out using ESI mode.

### Electrophysiology

Capped cRNA for the α3 and β4 subunits was made using the mMessage mMachine SP6 transcription kit *in vitro* (Thermo Fisher Scientific, Ambion, Austin, TX, USA). Then the cRNA was purified by using MEGAclear™ kit. Each *Xenopus* oocyte was injected with at least 20 ng of cRNA and incubated with ND96 buffer (96 mm NaCl, 5 mm HEPES, 2.0 mm KCl, 1.8 mm CaCl_2_ and 1.0 mm MgCl_2_, pH 7.0–7.5) and incubated at 17 °C. Two‐electrode voltage clamp recordings were performed in oocytes 2–4 days after cRNA injection at a holding potential of −70 mV. Pipets were pulled from borosilicate glass and filled with 3 m KCl. Membrane currents were recorded using an Axoclamp 900A amplifier (Molecular Devices Corp., Sunnyvale, CA, USA). The oocytes were gravity‐perfused in a recording chamber (50 μL) with ND96 containing 1 m atropine and 0.1 mg·mL^−1^ BSA at a rate of 2 mL·min^−1^. A 100 μm ACh pulse was applied for 1 s at 5 min intervals. All recordings were done at room temperature. To obtain estimates of potency, concentration–response curves were fitted to the data by the equation: response (%) = 100/[1 + ([toxin]/IC_50_)^*n*^], using prism 6.0 (GraphPad Software, Inc., San Diego, CA, USA).

## Conflict of interest

The authors declare no conflict of interest.

## Author contributions

PX, YW, DZ and SL conceived and designed this study; PX performed the major experiments and wrote the manuscript; PX and YX acquired the data; PX, YX, YL and SY analyzed and interpreted the data; YW and SL participated in critically discussing and revising the manuscript.
